# Barriers to medications for opioid use disorder in the court system: provider availability, provider “trustworthiness,” and cost

**DOI:** 10.1186/s40352-022-00188-4

**Published:** 2022-07-27

**Authors:** Fatema Z. Ahmed, Barbara Andraka-Christou, M.H. Clark, Rachel Totaram, Danielle N. Atkins, Brandon del Pozo

**Affiliations:** 1 School of Global Health Management & Informatics, University of Central Florida, 528 W Livingston St, FL 32801 Orlando, USA; 2 Department of Internal Medicine (Joint Secondary Appointment), University of Central Florida, 6850 Lake Nona Blvd, Orlando, FL 32827 USA; 3 Department of Learning Sciences & Educational Research, University of Central Florida, 12494 University Blvd, Orlando, FL 32816 USA; 4 Warren Alpert Medical School, Brown University, 222 Richmond St, Providence, RI 02903 USA

**Keywords:** Court, Buprenorphine, Methadone, Naltrexone, Trust, Cost, Barriers, Availability, Florida, Survey, Opioid, Provider

## Abstract

**Background:**

Medications for opioid use disorder (MOUD) significantly decrease mortality but courts rarely refer participants with opioid use disorder to MOUD providers. Previous qualitative work suggests routine court referrals to MOUD providers are more likely if court team members perceive providers as “trustworthy.” Court team members may also be less likely to refer participants to MOUD if they consider MOUD unaffordable, particularly in Florida, which has not expanded Medicaid. Our aims were to explore court team members’ 1) perceptions of availability of local trustworthy MOUD providers, 2) characteristics associated with perceptions of availability of local trustworthy MOUD providers, including beliefs about MOUD efficacy, and 3) perceptions of MOUD affordability.

**Methods:**

An online survey was distributed to all criminal problem-solving court and dependency court team members in Florida in 2019 and 2020. Likert scale questions assessed respondent agreement with statements about the availability of any MOUD providers, the availability of trustworthy MOUD providers, and the affordability of MOUD for court participants. An open-ended question explored MOUD barriers. Spearman’s rho, Friedman, Kruskal Wallis, and Mann-Whitney U tests were used for analyzing quantitative data and iterative categorization for qualitative data.

**Results:**

One hundred fifty-one respondents completed quantitative questions (26% response rate), and 42 completed the qualitative question. Respondents were more likely to agree that *local* MOUD providers are more available than *trustworthy* MOUD providers. Perceptions of trustworthy provider availability differed significantly by MOUD type and were associated with MOUD efficacy beliefs. Qualitative results suggest that MOUD providers offering counseling and individualized treatment are more trustworthy.

**Conclusions:**

Court team MOUD beliefs may influence their perceptions of providers, or negative experiences with providers may influence court team MOUD beliefs. Improving court team perceptions of local MOUD providers may be critical for facilitating court participant treatment access.

**Supplementary Information:**

The online version contains supplementary material available at 10.1186/s40352-022-00188-4.

## Introduction

In the US, criminal problem-solving courts, such as adult drug courts, veterans courts, mental health courts, driving under the influence courts, and juvenile courts, serve as an alternative to arrest/incarceration when a crime is related to a substance use disorder (SUD) or mental health disorder (National Association of Drug Court Professionals, 2015). Civil dependency courts, including general dependency courts, family dependency drug courts, and early childhood courts, seek to reunify parents with children whose custody was lost due to drug use (Center for Children and Family Futures and National Association of Drug Court Professionals, 2019). Criminal problem-solving courts and civil dependency courts mandate SUD treatment for participants with SUD, refer participants to treatment, and monitor treatment progress using an interdisciplinary team-based approach. Practices are generally similar across problem-solving courts, whether criminal or civil (Marlowe et al., 2016), except that criminal problem-solving courts can use jail time as sanctions to increase compliance with court requirements.

Problem-solving court teams typically include judges, court administrators (who oversee day-to-day operations of the court), court case managers, a treatment provider representative from a collaborating treatment agency, and other professionals. Voluntary national best practice standards recommend that problem-solving courts collaborate with only one or two behavioral health treatment agencies, with regular participation of treatment agency representatives (e.g., counselors) on the court team (National Association of Drug Court Professionals, 2015, 2018). Criminal problem-solving courts may include law enforcement, correction officials, and prosecutors on their team, while veterans courts typically include a veterans outreach specialist to facilitate referrals for treatment to the Veterans Health Administration (Andraka-Christou, 2017). While the court system employs judges, court administrators, and court case managers, other court team members may be used by other community agencies (e.g., probation officers by the department of corrections and the treatment provider representative by a community treatment agency).

Unfortunately, fewer than one in 20 justice-involved individuals with opioid use disorder (OUD) are referred to agonist medications for opioid use disorder (MOUD) (Krawczyk et al., 2017), which are the most effective treatments for OUD (Wakeman et al., 2020). Buprenorphine and methadone, both agonist medications, lower the rate of mortality by as much as 50% in people with OUD (Santo Jr. et al., 2021). Across criminal justice institutions, diversionary programs, like criminal problem-solving courts, are among the least likely to refer to agonist treatment. For example, in diversionary programs, only 2% of people with OUD are referred to agonist treatment, as compared to 3% from other courts, 5% from probation/parole, and 10% from prison (Krawczyk et al., 2017). It is unclear why problem-solving courts are less likely than other criminal justice institutions to refer individuals to agonist treatment, but low referrals are particularly concerning given that problem-solving courts are designed to facilitate SUD treatment and address underlying causes of drug-related behaviors (Krawczyk et al., 2017). Moreover, a recent Department of Justice ruling states that it is a violation of the American Disabilities Act to prohibit or limit the use of OUD treatment for individuals under court supervision (Department of Justice, 2022).

Some problem-solving courts have policies prohibiting the use of MOUD (Matusow et al., 2013), potentially explaining low MOUD utilization by court clients. For example, juvenile courts might have policies against referring adolescents to MOUD since the medications have not been approved for people under 18, even though studies demonstrate medication efficacy in adolescents (McCarty et al., 2021). Since courts typically operate autonomously, different courts in the same geographic area can have different policies and practices related to MOUD, unless restricted by state or federal law (Andraka-Christou et al., 2021). For example, federal law currently prohibits the receipt of grant funding by courts that ban MOUD utilization (U.S. Bureau of Justice Assistance, n.d.), and a few states have passed laws requiring courts to allow MOUD utilization (Andraka-Christou et al., 2022). Some national professional organizations, like the National Association of Drug Court Professionals, have passed voluntary best practice guidelines in an attempt to standardize treatment practices in problem-solving courts (National Association of Drug Court Professionals, 2018).

Several studies have also found negative attitudes toward agonist MOUD among court staff (Andraka-Christou, 2017; Andraka-Christou et al., 2019; Andraka-Christou & Atkins, 2020a, 2020b; Csete & Catania, 2013; Matusow et al., 2013). In contrast, court staff attitudes appear more favorable toward extended-release naltrexone (Andraka-Christou, 2017; Andraka-Christou et al., 2019; Andraka-Christou & Atkins, 2020a, 2020b; Csete & Catania, 2013; Matusow et al., 2013), an antagonist MOUD that lacks misuse potential but has lower efficacy in preventing overdose death (Wakeman et al., 2020). It is likely that court staff beliefs about MOUD influence court MOUD policies and referral practices (Andraka-Christou, 2017).

In addition to court policies, MOUD may be underutilized by court clients due to geographic disparities in the availability of MOUD. For example, even though most US counties now have at least one agonist MOUD provider (Andrilla & Patterson, 2021), such providers are less common in rural areas. Court clients in rural areas may also experience more transportation difficulties in accessing MOUD (Andrilla, Moore, Patterson, & Larson, 2019; Joudrey, Edelman, & Wang, 2019; Thomas, Van de Ven, & Mulrooney, 2020).

To develop more effective governmental policies and interventions for facilitating MOUD access among court participants, more information is needed about barriers to MOUD referrals in the court system, including how these barriers differ by court type and medication. For example, most studies to date on MOUD barriers in the court system have either not compared all three medications or have only focused on adult drug courts (Andraka-Christou, 2017; Csete & Catania, 2013; Fendrich & LeBel, 2019; Finigan et al., 2011; Gallagher et al., 2018; Gallagher et al., 2019; Hall et al., 2016; Krawczyk et al., 2017; Matusow et al., 2013; Robertson & Swartz, 2018; Substance Abuse and Mental Health Services Administration, 2014; Taxman & Bouffard, 2003), excluding other types of courts that regularly refer clients to SUD treatment (e.g., veterans courts, mental health courts, juvenile drug courts, and family dependency drug courts) (Matusow et al., 2013). Moreover, even though a lack of local MOUD providers is a known barrier to court referrals (Andraka-Christou, 2017; Csete & Catania, 2013; Matusow et al., 2013), prior studies have not disaggregated between the availability of *any* MOUD provider and availability of a MOUD provider whom court team members trust. Our study builds on prior work by examining court team members’ perceptions of the availability of trustworthy MOUD providers.

Research in the field of interorganizational relationships suggests that trust is one of several potential predictors of continuation and formation of interorganizational relationships (Nielsen, 2004). While several approaches to conceptualizing interorganizational trust exist, our research is guided by the general principle that interorganizational trust involves two key components: perception of a partner’s competence (e.g., technical skills, expertise, reliability) and integrity (e.g., motives, honesty, character) (Connelly et al., 2015). The role of trustworthiness in development and continuation of court-MOUD provider relationships remains poorly understood, although prior qualitative work suggests that court-MOUD provider relationships are unlikely to be established if court team members view local MOUD providers with distrust (Andraka-Christou, 2017). It is possible, for example, that court team members perceive local MOUD providers as lacking in competence or integrity, thereby hindering development of an interorganizational relationship.

It is also possible that court team members’ perceptions of the trustworthiness of an MOUD provider relate to the team members’ beliefs about the medication offered. For example, if a court team member believes methadone is inherently harmful, then they might also distrust methadone providers. To date, no study has examined the relationship between court team member perceptions of MOUD provider trustworthiness and team member beliefs about MOUD. Such information is necessary to inform the development of interventions facilitating relationships between court staff and MOUD providers, thereby potentially increasing referrals to MOUD treatment.

To help address these gaps, we used an online survey with an optional free-response text space to explore Florida criminal problem-solving court and civil dependency court team members’ perceptions of MOUD barriers. Specifically, we sought to (1) identify the relative frequency of different types of perceived barriers, comparing across medications, (2) explore the relationship between perceived MOUD barriers and beliefs about MOUD efficacy/safety, and (3) explore differences in perceived barriers by court type (i.e., criminal versus civil) and court role (e.g., judge, case manager). This research is part of a larger project examining MOUD barriers in the Florida court system (Andraka-Christou et al., 2020; Andraka-Christou et al., 2021; Andraka-Christou & Atkins, 2020a, 2020b).

## Methods

### Instrument development

We drafted survey questions about three types of barriers based on a literature review. Specifically, respondents were asked to indicate their agreement on a five-point Likert scale (strongly disagree, disagree, neutral, agree, strongly agree) with the following barrier statements: “No [medication] providers are located near our court,” “No trustworthy [medication] providers are located near our court,” and “Clients lack financial resources to pay for [medication] treatment.” These questions were asked separately for each of the three MOUD types.

Questions about barriers were related to the concept of self-efficacy: meaning, whether respondents felt they could refer clients to MOUD based on the availability of any providers, the availability of trustworthy providers, and whether clients referred to MOUD could afford it. The barrier questions were part of a larger survey we designed based on the theory of planned behavior (Ajzen, 1996), which posits that intentions to perform a behavior (e.g., to refer individuals to MOUD) are influenced by the actor’s beliefs, perception of social norms, and feelings of self-efficacy. Therefore, our survey also included questions about social norms related to MOUD and beliefs about MOUD safety and efficacy (modeled on questions from Matusow et al., 2013), with results from those questions and details of their development reported elsewhere (Andraka-Christou & Atkins, 2020a, 2020b; Matusow et al., 2013). See Additional file 1 for beliefs questions.

At the end of the survey, we asked an optional, open-ended question: “Is there anything else you would like the researchers to know about policies, attitudes, and barriers related to medication-assisted treatment (e.g., formulations of methadone, buprenorphine, naltrexone) for opioid use disorder?” During instrument development, we piloted the survey questions with a few court staff to obtain feedback regarding clarity of wording and relevance of questions. Staff recommended that we use the phrase “medication-assisted treatment” rather than “medications for opioid use disorder” and that we include brand names of medications along with generic names throughout the survey. The survey instrument also asked respondents to indicate their court type, court role (e.g., judge, case manager), and whether their court is entirely urban, mostly urban, completely rural, or mostly rural. Since some court team members work with more than one court, respondents were instructed to only answer questions concerning their primary court.

### Data collection

The online survey was distributed by the research team via Qualtrics (Qualtrics Experience Management Platform, 2018) to all criminal problem-solving and civil dependency judges and court team members (e.g., case managers, court administrators, probation officers, counselors, clinical case managers) in Florida twice, once in Summer 2019 and once in Summer 2020, using contact information obtained from a state court agency. Reminders were sent weekly for one month. Additionally, the state court agency sent recruitment messages to court team members directly via email, suggesting that anyone who had not yet received a message from the research team should contact the principal investigator if interested. No incentives were provided for survey completion. We collected data from a range of court roles because problem-solving courts are operated by interdisciplinary teams and from a range of courts because SUD services are commonly utilized by participants across different types of problem-solving courts (Strong et al., 2012).

### Ethics

This research was approved by the University of Central Florida’s Institutional Review Board, and each participant was provided with an explanation of research at the beginning of the survey.

### Quantitative data analysis

For analyses, we created two court type categories: criminal problem-solving courts (i.e., adult drug courts, veterans’ courts, juvenile courts, mental health courts, and DUI courts) and civil dependency courts (i.e., family dependency drug courts, early childhood courts, and general dependency courts). Due to the small cell sizes for some court roles, we created four categories of roles: court case managers, court administrators, judges, and others. We also categorized completely urban and mostly urban courts as urban, and we categorized completely rural and mostly rural courts as rural.

Descriptive statistics were used to examine the proportion of respondents who agreed with barrier statements and to summarize respondent characteristics. For the descriptive statistics, the Likert items were dichotomized into “agree” and “disagree.”

For inferential statistics, all five options were used from the Likert items. Associations between barriers and other factors were tested using non-parametric tests since all barriers were measured on an ordinal scale. A Friedman test was used to compare differences in beliefs between medications. A Kruskal Wallis test compared differences between team members’ roles on perceived MOUD barriers. A Mann-Whitney U test compared differences in MOUD barriers by court type and rurality.

We hypothesized that respondents would be more likely to believe a local provider is “trustworthy” if the respondent believes the medication provided by the provider is safe/effective. This hypothesis was based on the theory of stigma by association, wherein an individual (e.g., treatment provider) becomes stigmatized via associating with a stigmatized group (e.g., people with SUD) or stigmatized intervention (e.g., MOUD) (Green et al., 2021). We used Spearman’s rho to test correlations between beliefs about and perceived MOUD barriers.

### Qualitative data analysis

Free text responses (i.e., the qualitative data) of individuals who completed the survey in both 2019 and 2020 were removed before analysis. The remaining qualitative data were analyzed using iterative categorization (Neale, 2016). Iterative categorization involved the following steps. First, a codebook was created based on our research questions, a literature review, and a preliminary review of the data. Second, two researchers were assigned to code the data in Dedoose software Dedoose., 2018) using a consensus coding process, where they independently applied codes to each excerpt and then met to negotiate discrepancies. Multiple codes could be applied to an excerpt and the codebook was refined for clarity and relevance iteratively. Third, coded data were then exported to an Excel document, with different colors indicating different codes. Two researchers then independently labeled each exported datum with a summary statement (e.g., “providers who do not offer counseling are not considered trustworthy”). Fourth, they independently examined across all labels within a code to identify consistencies/inconsistencies and create a final domain summary for that code. Fifth, the researchers then met to discuss and negotiate differences in their domain summaries for each code. Lastly, the entire team met to discuss themes (i.e., overarching “threads”) across the domain summaries.

## Results

### Participant characteristics

Quantitative responses were obtained from 151 respondents who either completed the survey in Summer 2019 or Summer 2020. For those who completed the survey in both years, only their 2020 responses were analyzed. Our sample size is approximately 26% of all Florida criminal and problem-solving court team members, based on data received by our research team in 2019. Most respondents (58.9%) came from criminal problem-solving courts, primarily adult drug courts (35.8%). Approximately one-third (35.8%) came from civil courts, primarily general dependency courts (22.5%), and 5.3% were from “other” court types. Most respondents (41.1%) were judges, about one-quarter were court administrators (27.8%), and about one-tenth were court case managers (11.3%). The remainder included probation officers, counselors, clinical case managers, and other court team members. Most respondents said their court was in an urban or mostly urban area (67.5%), and most had a graduate degree (66.4%) (see Table 1). Open-ended responses were analyzed for 42 respondents.

**Table 1:** Sample characteristics

Court Type	Percentage
Criminal Problem-Solving Courts	58.9%
Adult Drug Court	35.8%
Veterans Court	9.3%
Juvenile Drug Court	4.0%
DUI Court	0.7%
Other criminal problem-solving court	9.3%
Civil Courts	35.8%
General Dependency Court	22.5%
Early Childhood Court	6.6%
Family Dependency Drug Court	2.6%
Other civil court	4.0%
Other court type	5.3%
**Court Role**	
Court case manager	11.3%
Court administrator	27.8%
Judge	41.1%
Other role	19.8%
**Other Characteristics**	
Female	74.7%
Urban or mostly urban area	67.5%
**Observations**	151

### Quantitative results

#### Availability of MOUD providers near the court

**Fig. 1:**
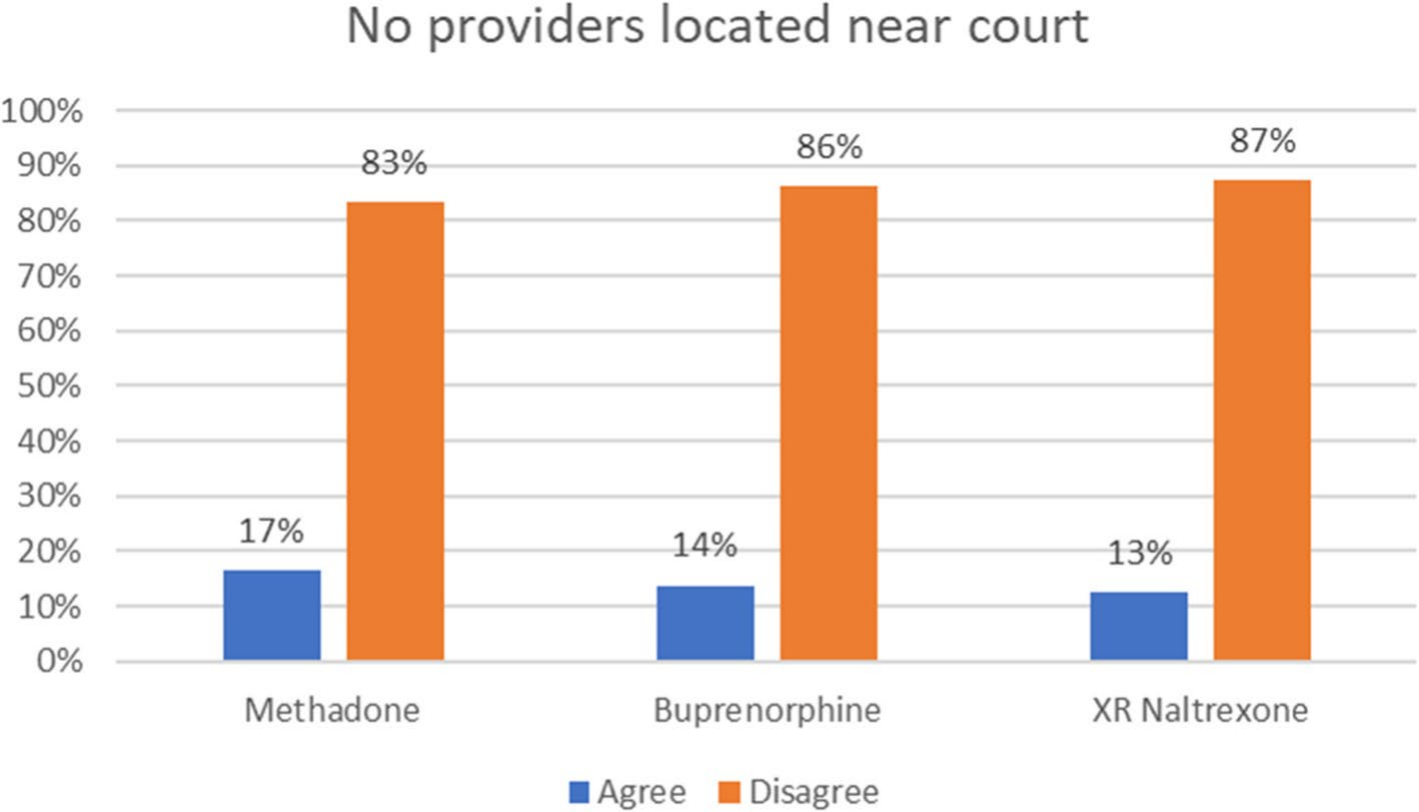
Response to Lack of MOUD Providers Near the Court by MOUD Type

Figure 1 shows the percentage of respondents who agreed or disagreed with the statement that no providers were located near the court by MOUD type. Less than 20% of respondents agreed that any providers were located near the court (see Fig. 1).

#### Availability of trustworthy MOUD providers near the court

**Fig. 2:**
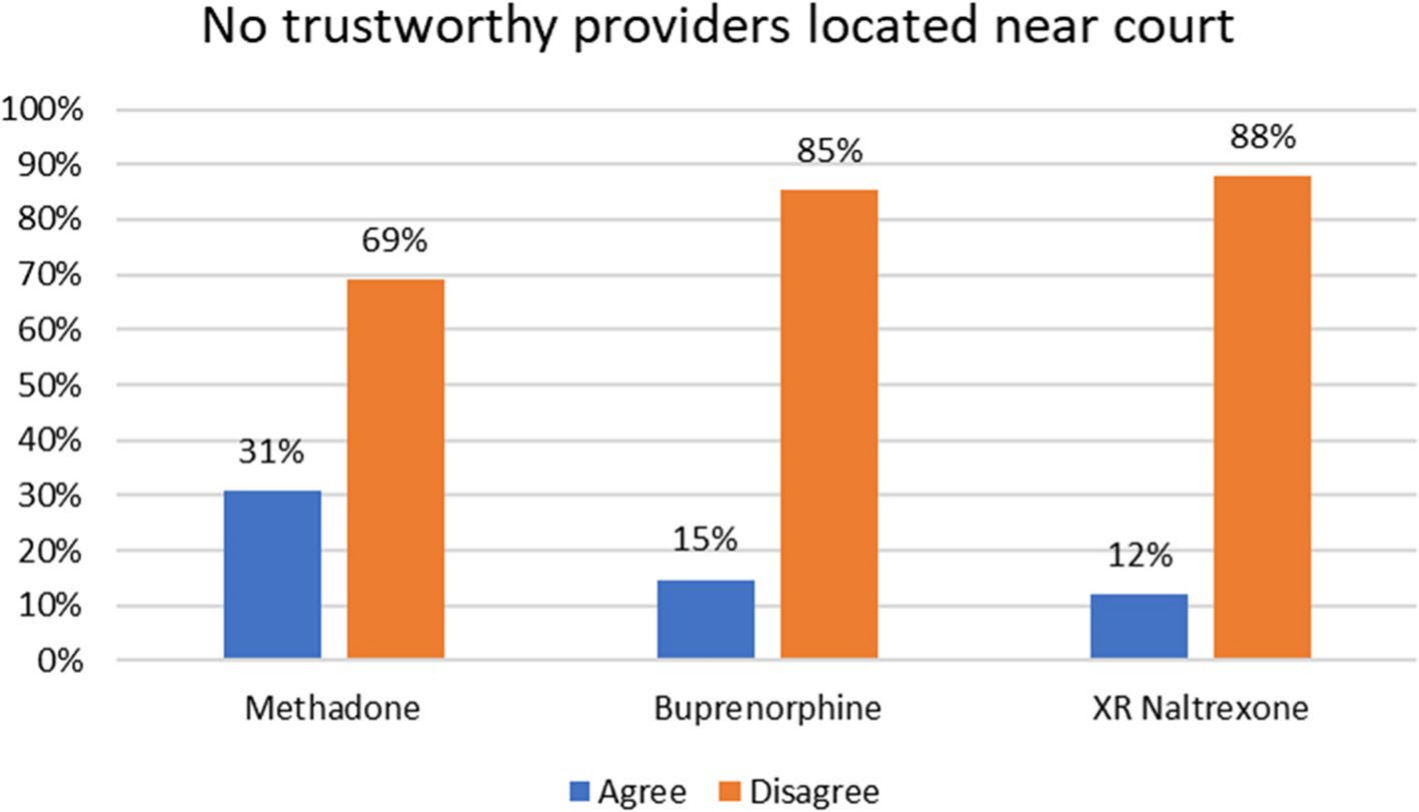
Response to Lack of Trustworthy MOUD Providers Near the Court by MOUD Type

Figure 2 shows the percentage of respondents who agreed or disagreed with the statement that there were no trustworthy providers near the court by MOUD type. About 30% of respondents agreed that there were no trustworthy methadone providers located near the court, compared to 15% that agreed there were no trustworthy buprenorphine providers near the court and 12% who agreed there were no trustworthy XR-naltrexone providers near the court (see Fig. 2).

#### Client ability to pay for MOUD

**Fig. 3:**
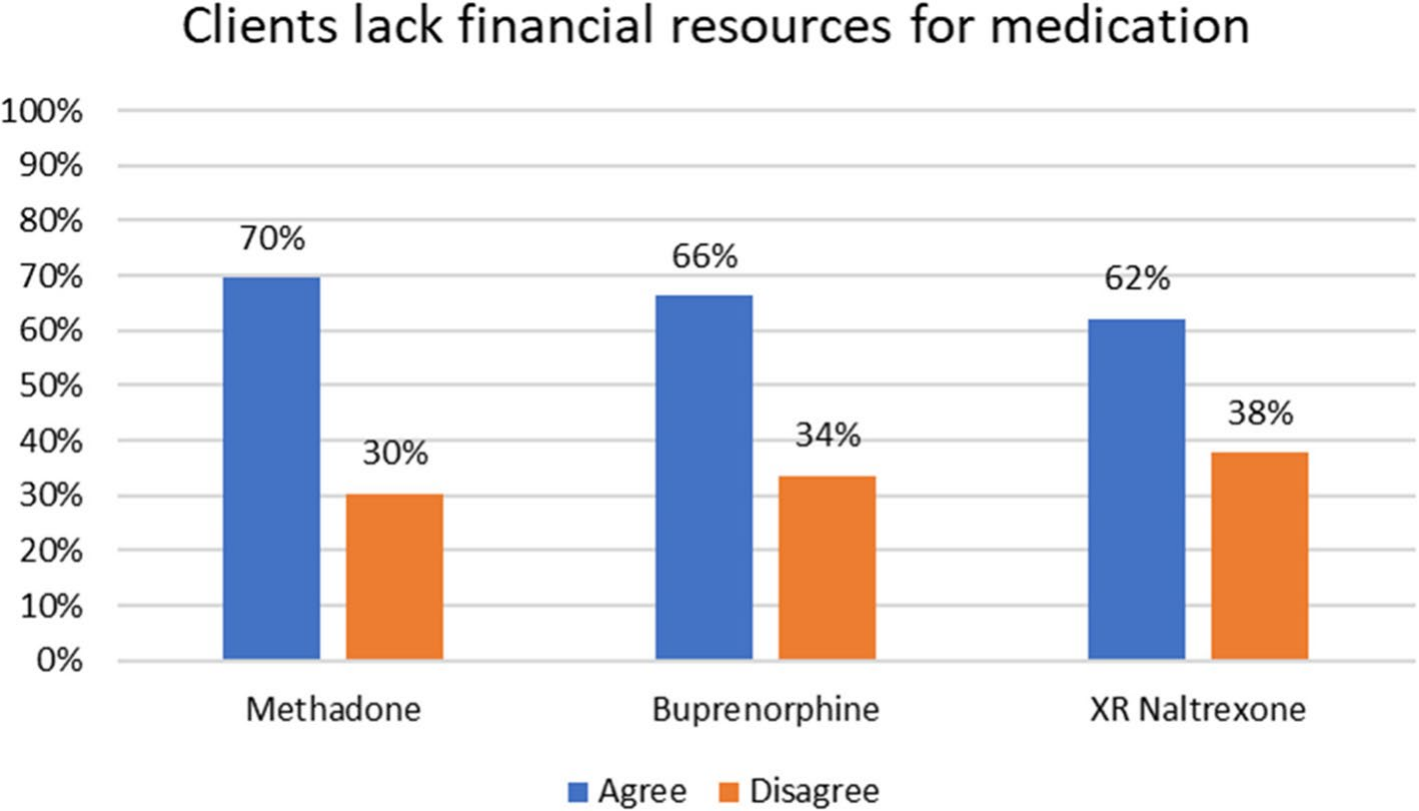
Response to Clients Lack of Financial Resources to Pay for MOUD by MOUD Type

Figure 3 shows the percentage of respondents that agreed or disagreed with the statement that clients lack financial resources to pay for MOUD by MOUD type. Most respondents agreed that clients lack financial resources to pay for methadone (70%), buprenorphine (66%), and XR-Naltrexone (62%) (see Fig. 3).

#### Relationships between perceptions of available trustworthy MOUD providers and beliefs about MOUD efficacy/safety

Five of the 11 belief statements about efficacy/safety were significantly correlated with responses to methadone barriers questions, eight with responses to buprenorphine barriers questions, and eight with responses to XR-naltrexone questions. Those who believed that no *trustworthy* MOUD provider was located near the court were *less* likely to agree that methadone, buprenorphine, and XR-naltrexone reduce relapse, reduce crime and incarceration, and should be used to maintain clients with OUD (see Table 2). They were also *less* likely to agree that XR-naltrexone reduces or blocks the effects of heroin (See Table 2). Respondents who believed no *trustworthy* MOUD provider was located near the court were *more* likely to agree that buprenorphine and XR-naltrexone reward drug users, prolong addiction, interfere with one’s ability to drive a car and that it is difficult for a parent to regain custody of a child while being treated with either medication (see Table 2). Additionally, they were *more* likely to agree that buprenorphine providers should have a titration plan for each patient. These results highlight some of the significant relationships, others can be found in Table 2.

**Table 2:** Spearman’s Rho between each belief about MOUD ^a^ and its barriers

Belief	No MOUD providers are located near our court			No trustworthy MOUD providers are located near our court			Clients lack financial resources to pay for MOUD treatment		
	M	B	N	M	B	N	M	B	N
MOUD reduces relapse	−.15	−.13	−.38***	−.41***	−.35***	−.50***	−.05	.01	.02
MOUD reduces crime and re-incarceration	−.12	−.20*	−.33***	−.30***	−.36***	−.37***	.05	.11	−.07
MOUD rewards criminals for being drug users	.10	.17	.19*	.12	.20*	.25*	−.02	−.04	−.09
MOUD prolongs addiction	−.05	.15	.26**	.14	.22*	.42***	−.03	−.05	−.05
MOUD should be used to maintain clients who have opioid use disorder	−.09	−.22*	−.28**	−.23*	−.21*	−.32***	.05	.14	.19
MOUD is more effective than non-pharmacological approaches (e.g., counseling) to retaining clients in treatment	−.15	−.08	−.04	−.14	−.01	−.09	−.04	.05	.00
MOUD interferes with one’s ability to drive a car	.01	.24*	.27*	.16	.24*	.33**	−.01	−.03	−.21
MOUD reduces or blocks the effect of heroin	.23*	−.12	−.27**	.02	.01	−.24*	.01	.20	−.03
In Florida, it is difficult for a parent to regain custody of a child while the parent is treated with MOUD	.17	.26*	.30**	.16	.27*	.39***	.37***	.05	.15
People should be allowed to access MOUD without counseling	.05	−.01	.08	−.15	−.10	.04	−.03	.09	.03
MOUD prescribers should have a titration plan for each patient	.04	.16	.05	.02	.23*	.13	.12	.08	.13

*Abbreviation*: *M* methadone, *B* buprenorphine, *N* naltrexone

* *p* < .05, ** *p* < .01, *** *p* < .001

#### Differences between medications for each barrier

A Friedman’s test for related samples examined differences in perceptions of barriers by type of medication. We did not find a significant difference by type of medication with respect to whether any MOUD provider is located near the court or based on clients’ ability to pay for treatment. However, the perception of whether a *trustworthy* provider was near the court significantly depended on the type of medication, c^2^(2) = 35.550, *p* < .001, *N* = 95. Respondents were more likely to indicate that trustworthy XR-naltrexone (*M*_*rank*_ = 1.77) providers were near the court than trustworthy methadone (*M*_*rank*_ = 2.27) providers. However, there was no difference between having trustworthy buprenorphine providers near the court (*M*_*rank*_ = 1.96) as compared to either trustworthy methadone or XR-naltrexone providers.

#### Differences in barrier perceptions by team member role

A Kruskal Wallis test was run to compare the differences between team members’ roles on perceptions of MOUD barriers. We did not find any significant differences by role. Table 3 includes significance tests for each barrier.

**Table 3:** Differences in MOUD barriers by court role, type, and urbanity

	Role	Type			Urbanity		
Barrier	***H***	***U***	Mean Rank for Criminal Courts	Mean Rank for Family Courts	***U***	Mean Rank for Rural Courts	Mean Rank for Urban Courts
No methadone providers are located near our court	8.81	1487.00	57.83	62.42	1108.00***	78.34	57.09
No buprenorphine providers are located near our court	9.56	1101.50	52.69	61.53	928.50***	72.86	53.55
No naltrexone providers are located near our court	9.07	776.00***	48.71	66.97	803.50***	72.29	52.45
No trustworthy methadone providers are located near our court	9.09	1276.00	53.49	54.92	1112.00	65.30	55.06
No trustworthy buprenorphine providers are located near our court	11.03	934.50	50.13	61.30	925.00*	67.10	53.51
No trustworthy naltrexone providers are located near our court	14.07	587.00***	45.43	67.54	751.00**	68.19	50.66
Clients lack financial resources to pay for methadone treatment	11.83	1794.00**	61.00	44.65	857.00**	73.03	51.95
Clients lack financial resources to pay for buprenorphine treatment	14.17	1447.00	56.17	49.89	1017.00*	67.59	53.21
Clients lack financial resources to pay for naltrexone treatment	6.798	1080.00	51.50	54.75	950.00	64.24	53.09

*Abbreviation*: *H* Kruskal Wallis value, *U* Mann-Whitney U value

* *p* < .05, ** *p* < .01, *** *p* < .001

#### Differences in barrier perceptions by court type

A Mann-Whitney U test compared the differences in MOUD barriers by court type (criminal problem-solving court vs. civil dependency court). Type of court was significantly related to three of the nine barriers. Respondents in criminal problem-solving courts were *less* likely to agree that (a) no XR-naltrexone provider was near their court and (b) no *trustworthy* XR-naltrexone provider was near their court than respondents in civil dependency courts (see Table 3). Respondents in criminal problem-solving courts were also *more* likely to agree that clients could not afford methadone than those in civil dependency courts (see Table 3).

#### Differences in barrier perceptions by urbanity/rurality

A Mann-Whitney U test compared MOUD barriers by urban versus rural court location. Court location was associated with seven of the nine barriers. As compared to respondents in urban courts, respondents in rural courts were significantly more likely to agree that (a) no methadone, buprenorphine, or XR-naltrexone providers were near their courts; (b) no *trustworthy* buprenorphine or XR-naltrexone providers were near their courts; and (c) clients could not afford methadone or buprenorphine (see Table 3).

### Qualitative results

We identified four qualitative themes regarding MOUD barriers: (a) cost is a barrier to MOUD access; (b) lack of court team MOUD education is a barrier to facilitating MOUD; (c) many sources of stigma exist, including outside of the court systems; and (d) court-provider relationships can facilitate MOUD access or reinforce MOUD stigma. See Table 4 for example quotes for each theme.

**Table 4:** Example quotations from free text responses

Theme	Example Quotes
*MOUD cost is a barrier to client access*	*• “From the dependency bench I have very little control over the specific drug treatment offered to the parents. I can learn how it works but if the department doesn’t offer any treatment of that type or pay for it this type of treatment is not going to happen.” - R*espondent 30 *• “Price dictates treatment” -* Respondent 37 *• “We have only had funding for Vivitrol and the flow of funds for methadone and suboxone are just now coming through.” -* Respondent 1
*Lack of MOUD education is a barrier to courts facilitating MOUD for clients*	*• “I know nothing of the side effects of the treatment and whether or not it would impair a parent’s ability to work, pass drug tests, and parent young children.” -* Respondent 30 *• “We were taught that methadone and suboxone were addictive when we were convinced Vivitrol was better. Now, the education and feelings are that all 3 are needed, all will be funded and that vivitrol is not appropriate in some situations. We all need to get on the same page and ensure that the proper training is given and that it is not steered by lobbying and big pharma for their own products and that our treatment providers prescribe the one that is best suited for each client!” -* Respondent 1 *• “Education to inform that M.A.T. should be viewed as medication (for some a lifelong medication) as opposed to a drug.” -* Respondent 31
*Many source of stigma toward MOUD, including outside of the court system*	*• “Individuals in recovery who are NOT using MAT may pass judgment to those who are using it. I have heard from several clients that use MAT that they feel ashamed or uncomfortable admitting that they are on MAT to others in recovery, specifically at support group meetings.” -* Respondent 45 *• “Difficult to find sober living homes and [peer support] fellowships that allow/support MAT” -* Respondent 18
*Court-provider relationship relationships can facilitate MOUD access or reinforce MOUD stigma*	*• “Collaborating with providers who are trustworthy is very difficult. We have a few identified but they cannot meet the full need of the courts let alone the community. Access to these medications in pharmacies play a role in the ability of community clients to get what they need in regards to treatment and the pharmacists are looking for treatment plans and legitimate providers as well. We still have a number of pill mills in our area and questionable prescribers, many have been previously fined by the licensing board. How do we identify and collaborate with other prescribers willing to work with us?” -* Respondent 25 *• “Due to no reputable methadone providers in our area, MAT programs have had a negative connotation previously.” -* Respondent 24 *• “…patients have been more successful when receiving therapy along with Naltrexone than therapy with the other forms of MAT as they are often still taking medications upon completion of the program and end up without any way of paying for the medications so they revert to prior habits or purchasing off the street which is dangerous.” -* Respondent 42

#### Cost is a barrier to MOUD access

Respondents described clients’ lack of financial access to MOUD, courts’ inability to help clients pay for MOUD in general, and courts’ inability to help clients pay for the type of MOUD the client prefers. Respondents explained that the state of Florida sometimes only funded one or two types of MOUD for court clients, limiting the type of MOUD accessible to clients. For example, Participant 1 stated, “... I have very little control over the specific drug treatment offered to the parents. I can learn how it works but if the department doesn’t offer any treatment of that type or pay for it this type of treatment is not going to happen.” Additionally, a perception existed that the priorities of local agencies that distribute state funding to SUD treatment providers do not necessarily align with those of the court and that these agencies sometimes allocate resources based on preferential treatment of certain SUD treatment providers rather than client needs.

#### Lack of MOUD education is a barrier to courts facilitating MOUD for clients

The court team desired more training and education about the purpose, efficacy, and physiological mechanisms of MOUD, which could help address MOUD stigma. Also, the need for education from reliable sources was indicated as opposed to pharmaceutical companies. For example, Participant 2 explained that the trainings received by court team members about MOUD have been inconsistent, leading to confusion about which MOUD is most effective (e.g., methadone versus XR-naltrexone), stating, “We were taught that methadone and suboxone were addictive when we were convinced Vivitrol was better. Now, the education and feelings are that all three are needed, all will be funded, and that Vivitrol is not appropriate in some situations...”

#### Many sources of MOUD stigma exist, including outside of the court system

Some respondents indicated their own stigma toward MOUD in open-ended responses by highlighting misuse/diversion concerns. For example, Participant 3 stated, “My perception is that the methadone treatment is overused and abused the addicts that it really does not address the addictive behavior. It just eases the pain of the desire. This is just my perception based on seeing the same people over and over in dependency and criminal court.” Respondents described other team members in the court system and people within the recovery community as having negative beliefs about MOUD. Respondents noted that MOUD stigma causes shame for court clients and prevents treatment retention.

#### Court-provider relationships can facilitate MOUD access or reinforce MOUD stigma

Respondents described their relationship with SUD treatment providers as a barrier to client MOUD access. Respondents identified a shortage of local MOUD providers, which prevents courts from establishing and maintaining relationships with MOUD providers. Respondents described providers who do not offer behavioral therapy or individualized care as “untrustworthy,” “disreputable,” and “pill mills.” For example, Participant 4 stated, “Our area has one ‘methadone clinic’ that only provides methadone for opioid addiction, and one substance abuse provider that provides Vivitrol (at no cost to clients). Suboxone is available only from a few physicians in our area. I see mostly methadone treatment, and it is the exact same dose, etc. for nearly every patient.” Regarding providers and behavioral therapies, Participant 5 stated, “Patients have been more successful when receiving therapy along with Naltrexone than therapy with the other forms of MAT as they are often still taking medications upon completion of the program and end up without any way of paying for the medications, so they revert to prior habits or purchasing off the street which is dangerous.” Respondents also expressed a desire for help in identifying trustworthy MOUD providers.

## Discussion

MOUD is the treatment for OUD most likely to reduce overdose and addiction-related hospitalizations (Wakeman et al., 2020), but it is underutilized by court-involved individuals with OUD (Krawczyk et al., 2017). We examined court team member perceptions of three types of MOUD barriers: client inability to pay, lack of availability of a MOUD provider, and lack of availability of a “trustworthy” MOUD provider. Among these three barriers, client inability to pay was most common, with at least 60% of respondents indicating that clients could not afford MOUDs.

Unlike most US states, Florida has not expanded Medicaid (Kaiser Family Foundation, 2020), a government health insurance program for low-income individuals. Instead, Florida has used targeted grants and legislative mechanisms to pay for specific types of treatments for defined populations. For example, in recent years, the Florida legislature has appropriated funding specifically for XR-naltrexone payments for justice-involved populations (Vivitrol Program History, 2020), likely explaining our findings that XR-naltrexone was perceived as more financially accessible than methadone or buprenorphine, even though buprenorphine and methadone (but not XR-naltrexone) have generic, off-patent formulations. We also found that respondents from rural courts were likelier than those from urban areas to indicate client MOUD cost barriers. Clients in rural areas may have lower incomes or less health insurance access than those in urban areas. Alternatively, courts and treatment providers in rural areas may lack the staff capacity to apply for the government grants that often fund treatment in Florida.

We found that while most respondents believed MOUD providers were available in their area, fewer felt that *trustworthy* MOUD providers were available – thereby, indicating that not all MOUD providers are deemed trustworthy by court staff. A previous qualitative study suggests that some court clients seeking to use MOUD from treatment agencies/providers with whom the court does not have an existing collaboration must first prove that the MOUD provider is “trustworthy” (e.g., one that monitors treatment effectively and provides comprehensive behavioral health services) (Andraka-Christou, 2017) – a task that may be difficult for vulnerable populations and could limit MOUD access to only those clients who are most persuasive. In line with our expectations, respondents were significantly more likely to perceive a lack of available trustworthy methadone providers than trustworthy XR-naltrexone providers, even after conditioning on the availability of local MOUD providers. The qualitative data suggests that trainings provided by the manufacturer of XR-naltrexone to court staff may have affected the perceived availability of trustworthy XR-naltrexone providers in the area.

Our study is the first to examine the relationship between perceptions of the availability of trustworthy MOUD providers and beliefs about MOUD. As expected, we found the perceptions of the availability of trustworthy MOUD providers were significantly associated with respondents’ beliefs about the safety/efficacy of the medication provided. For example, respondents who believed that buprenorphine prolongs addiction were more likely to believe that no trustworthy buprenorphine providers existed in the area. It is possible that beliefs about medications influence the perception of providers of the medication. Alternatively, experiences with treatment providers could affect court team members’ perceptions of the medications offered. If the latter is true, then it is possible that state policy initiatives to connect court team members to MOUD providers who have a reputation for integrity and competence (two components of trust) could lead to increased positive attitudes about the medications. Significantly more research, however, is needed to understand the mechanisms by which court team members’ beliefs about MOUD change.

The National Association of Drug Court Professionals, a standard-setting body for problem-solving courts, encourages the inclusion of treatment providers on the court team and recommends that courts only form interagency relationships with a few treatment providers (National Association of Drug Court Professionals, 2015). Since it is likely that courts will only form relationships with MOUD providers whom the team deem “trustworthy,” significantly more qualitative data are needed to accurately understand and operationalize the variable that describes trustworthiness in this context, as well as how to address concerns of lack of trustworthiness. Open-ended survey responses from our study helped illuminate characteristics that court team members associate with non-trustworthy as compared to trustworthy providers. For example, terms related to a provider’s integrity, such as “disreputable” and “pill mill,” were used to describe untrustworthy providers, while MOUD providers who recommended mental health counseling were considered more trustworthy.

Our study has several limitations. Our sample is not representative, and those who opted into the survey, knowing that it was about MOUD, may have been more likely to have pre-existing views toward MOUD or experience with MOUD in their court, resulting in perceptions of barriers that differ from those with no MOUD experience. Our data included both perspectives from 2019 and 2020, and it is possible that MOUD accessibility differed somewhat in both years (e.g., as new providers obtained buprenorphine waivers in the area). Moreover, our data collection coincided with the onset of the COVID-19 pandemic, which may have impacted access to treatment providers. It should also be noted that we had a low response rate of 26%, which is, in fact, an estimate because while we have statewide criminal and civil problem-solving court team data from 2019, we do not have it from 2020. Additionally, for those who took the survey in both 2019 and in 2020, we excluded their 2019 responses and only analyzed their 2020 responses to reflect their most recent views. Our survey only included questions about barriers that our team deemed most salient based on the literature and prior research experience, while we subsequently identified additional barriers in our qualitative results. We aggregated court types into criminal problem-solving courts and civil dependency courts due to small cell sizes for individual court types; thus, we are unable to examine underlying heterogeneity in court types due to sampling size limitations. The data were collected from court team members who may have inaccurate views about whether MOUD providers exist in their locality and whether court clients can afford MOUD. Nevertheless, we believe even inaccurate perceptions are important because they could influence court MOUD referrals insofar as perceptions guide behavior. Lastly, our study asked questions about local MOUD providers only, but problem-solving courts could benefit from forming relationships with providers further away from the court as well.

## Conclusion

Given the lifesaving potential of MOUD and the reliance of problem-solving courts on collaborating treatment providers, the problem-solving court field must develop interventions to facilitate interorganizational relationships between courts and MOUD providers. One approach could involve state-level requirements for courts to collaborate with MOUD providers. Indeed, in some states laws have been recently enacted to guide and promote the use of MOUD in problem-solving courts as a standard of care for clients who demonstrate the need for it (Andraka-Christou et al., 2022). State-level policies encouraging the use of MOUD not only acknowledge its effectiveness but may also help improve individual court team members’ beliefs about MOUD by creating clear performance expectations across otherwise autonomous courtrooms. Even with the development of court-MOUD provider relationships, however, it is possible for MOUDs’ cost to remain a barrier, particularly in states that have not expanded Medicaid. Insurance expansion and grants to courts to cover the cost of all forms of MOUD could help address cost barriers.

## Supplementary Information

**Additional file 1.** Relevant survey questions.

## Data Availability

Deidentified survey data, with small cell sizes redacted, is available from the corresponding author by request.

## Abbreviations

MOUD: Medications for Opioid Use Disorder
OUD: Opioid Use Disorder
SUD: Substance Use Disorder
XR-naltrexone: Extended-release Naltrexone

## References

[CR1] Ajzen I (1996). Behavioral interventions based on the theory of planned behavior. Organizational Behavior and Human Decision Processes.

[CR2] Andraka-Christou B (2017). What is treatment for opioid addiction in problem-solving courts? A study of 20 Indiana drug & veterans courts. CRCL.

[CR3] Andraka-Christou B, Atkins D (2020). Beliefs about medications for opioid use disorder among Florida criminal problem-solving court & dependency court staff. The American Journal of Drug and Alcohol Abuse.

[CR4] Andraka-Christou, B., & Atkins, D. N. (2020b). Whose opinion matters about medications for opioid use disorder? A cross-sectional survey of social norms among court staff. *Substance Abuse*, *1-16*. 10.1080/08897077.2020.1846666. 10.1080/08897077.2020.1846666 33284059

[CR5] Andraka-Christou B, Clark MH, Atkins DN, Del Pozo B (2021). Criminal problem-solving and civil dependency court policies regarding medications for opioid use disorder. Substance Abuse.

[CR6] Andraka-Christou B, Gabriel M, Madeira J, Silverman RD (2019). Court personnel attitudes towards medication-assisted treatment: A state-wide survey. Journal of Substance Abuse Treatment.

[CR7] Andraka-Christou B, Nguyen T, Bradford DW, Simon K (2020). Assessing the impact of drug courts on provider-directed marketing efforts by manufactures of medications for the treatment of opioid use disorder. Journal of Substance Abuse Treatment.

[CR8] Andraka-Christou B, Randall-Kosich O, Golan M, Totaram R, Saloner B, Gordon AJ, Stein BD (2022). A national survey of state laws regarding medications for opioid use disorder in problem-solving courts. Health Justice.

[CR9] Andrilla, C. H. A., & Patterson, D. G. (2021). Tracking the geographic distribution and growth of clinicians with a DEA waiver to prescribe buprenorphine to treat opioid use disorder. *The Journal of Rural Health.*10.1111/jrh.12569. 10.1111/jrh.12569 33733547

[CR10] Center for Children and Family Futures and National Association of Drug Court Professionals (2019). *Family treatment court best practice standards* (Supported by Grant #2016-DC-BX-K003 awarded by the Office of Juvenile Justice and Delinquency Prevention, Office of Justice Programs, U.S. Department of Justice.).

[CR11] Connelly BL, Crook R, Combs JG, Ketchen DJ, Aguinis H (2015). Competence- and integrity-based trust in interorganizational relationships: Which matters more?. Journal of Management.

[CR12] Csete, J., & Catania, H. (2013). Methadone treatment providers’ views of drug court policy and practice: A case study of New York state. *Harm Reduction Journal*, *10*(35), 1–9. 10.1186/1477-7517-10-35 PMC4176483 24308548

[CR13] Dedoose. (2018). Web application for managing, analyzing, and presenting qualitative and mixed method research data. Los Angeles: SocioCultural Research Consultants, LLC Available at www.dedoose.com.

[CR14] Department of Justice (2022). Justice Department finds that Pennsylvania courts discriminated against people with opioid use disorder.

[CR15] Fendrich M, LeBel TP (2019). Implementing access to medication assisted treatment in a drug treatment court: Correlates, consequences, and obstacles. Journal of Offender Rehabilitation.

[CR16] Finigan MW, Perkins T, Zold-Kilbourn P, Parks J, Stringer M (2011). Preliminary evaluation of extended-release naltrexone in Michigan and Missouri drug courts. Journal of Substance Abuse Treatment.

[CR17] Gallagher JR, Wahler EA, Lefebvre E, Paiano T, Carlton J, Miller JW, Woodward J (2018). Improving graduation rates in drug court through employment and schooling opportunities and medication-assisted treatment (MAT). Journal of Social Service Research.

[CR18] Gallagher JR, Wahler EA, Minasian RM, Edwards A (2019). Treating opioid use disorders in drug court: Participants’ views on using medication-assisted treatments (MATs) to support recovery. International Criminal Justice Review.

[CR19] Green B, Smith K, Schmitt A, Krane K (2021). The role of stigma in referrals for medication for opioid use disorder: Three case studies in Montan.

[CR20] Hall MT, Wilfong J, Huebner RA, Posze L, Willauer T (2016). Medication-assisted treatment improves child permanency outcomes for opioid-using families in the child welfare system. Journal of Substance Abuse Treatment.

[CR21] Kaiser Family Foundation (2020). *Status of state Medicaid expansion decisions: Interactive map*. Kaiser Family Foundation Retrieved January 27, 2021 from https://www.kff.org/medicaid/issue-brief/status-of-state-medicaid-expansion-decisions-interactive-map/#:~:text=To%20date%2C%2039%20states%20(including,available%20in%20a%20table%20format.

[CR22] Krawczyk N, Picher CE, Feder KA, Saloner B (2017). Only one in twenty justice-referred adults in specialty treatment for opioid use receive methadone or buprenorphine. Health Affairs.

[CR23] Marlowe DB, Hardin CD, Fox CL (2016). Painting the current picture: A national report on drug courts and other problem-solving courts in the United States.

[CR24] Matusow H, Dickman SL, Rich JD, Fong C, Dumont DM, Hardin C, Marlowe D, Rosenblum A (2013). Medication assisted treatment in US drug courts: Results from a nationwide survey of availability, barriers and attitudes. Journal of Substance Abuse Treatment.

[CR25] McCarty, D., Chan, B., Buchheit, B. M., Bougatsos, C., Grusing, S., & Chou, R. (2021). Effectiveness of and access to medications for opioid use disorder for adolescents and young adults: A scoping review. *Journal of Addiction Medicine*. 10.1097/ADM.0000000000000898. 10.1097/ADM.0000000000000898 PMC8761222 34282085

[CR26] National Association of Drug Court Professionals (2015). Adult drug court best practice standards volume II National Association of drug court professionals.

[CR27] National Association of Drug Court Professionals (2018). ADULT DRUG COURT BEST PRACTICE STANDARDS volume I text revision.

[CR28] Neale J (2016). Iterative categorization (IC): A systematic technique for analysing qualitative data. Addiction.

[CR29] Nielsen, B. (2004). The role of Trust in Collaborative Relationships: A multi-dimensional approach. *M@n@gement*, *7*(3). 10.3917/mana.073.0239.

[CR30] Qualtrics Experience Management Platform. (2018). (Version 1.3). Provo, Utah, USA. Available at https://www.qualtrics.com.

[CR31] Robertson AG, Swartz MS (2018). Extended-release naltrexone and drug treatment courts: Policy and evidence for implementing an evidence-based treatment. Journal of Substance Abuse Treatment.

[CR32] Santo T, Clark B, Hickman M, Grebely J, Campbell G, Sordo L, Chen A, Tran LT, Bharat C, Padmanathan P, Cousins G, Dupouy J, Kelty E, Muga R, Nosyk B, Min J, Pavarin R, Farrell M, Degenhardt L (2021). Association of opioid agonist treatment with all-cause mortality and specific causes of death among people with opioid dependence: A systematic review and meta-analysis. JAMA Psychiatry.

[CR33] Strong SM, Rantala RR, Kyckelhahn T (2012). Census of problem-solving courts, 2012 summary.

[CR34] Substance Abuse & Mental Health Services Administration (2014). Adult drug courts & medication-assisted treatment for opioid dependence.

[CR35] Taxman FS, Bouffard JA (2003). Substance abuse counselors’ treatment philosophy and the content of treatment services provided to offenders in drug court programs. Journal of Substance Abuse Treatment.

[CR36] U.S. Bureau of Justice Assistance. Medication-assisted Treatment. n.d. https://bja.ojp.gov/sites/g/files/xyckuh186/files/media/document/adc-faq-medication-assisted-treatment.pdf

[CR37] Vivitrol Program History (2020). Florida alcohol and drug abuse association and Florida behavioral health association.

[CR38] Wakeman SE, Larochelle MR, Ameli O, Chaisson CE, McPheeters JT, Crown WH, Azocar F, Sanghavi DM (2020). Comparative effectiveness of different treatment pathways for opioid use disorder. JAMA Network Open.

