# Supplementary material for: Barriers to medications for opioid use disorder in the court system: provider availability, provider “trustworthiness,” and cost
Source: Health Justice. 2022 Jul 27;10:24. doi: 10.1186/s40352-022-00188-4 (PMC9327334; doi:10.1186/s40352-022-00188-4)
Supplement: Supplementary file 1 — Additional file 1. Relevant survey questions. [file 40352_2022_188_MOESM1_ESM.docx]

**Additional file 1:**

Relevant survey questions

Q1 Among the following types of courts, in which one do you currently spend the MOST time as a staff member or team member?

- Adult drug court (1)
- Veterans Court (2)
- Juvenile drug court (3)
- Family dependency drug court (4)
- General dependency court (5)
- Early Childhood Court (6)
- DUI court (7)
- I choose not to answer (8)
- Other (9) ________________________________________________

Q2 Which of the following best describes your role on the court staff or court team? Select only one.

- Judge (1)
- Judicial Assistant (18)
- Court administrator/manager/coordinator (2)
- Court case manager (3)
- Clinical case manager (4)
- Substance use disorder counselor (5)
- Mental health counselor (6)
- Physician, nurse practitioner, or physician assistant (7)
- Veterans Justice Outreach Specialist (8)
- Department of Children and Families attorney (9)
- Defense attorney (10)
- Prosecutor (11)
- Police Officer (12)
- Probation or Parole officer (13)
- Peer support of parent partner (14)
- Clerk of Court (15)
- I choose not to answer (16)
- Other (17) ________________________________________________

Q4 To what extent do you agree with the statements below about **methadone** provided by a health care practitioner for opioid use disorder treatment?

|  | Strongly disagree (1) | Somewhat disagree (2) | Neither Agree nor disagree (3) | Somewhat agree (4) | Strongly agree (5) | I don't know (6) | I choose not to answer (7) |
| --- | --- | --- | --- | --- | --- | --- | --- |
| Methadone reduces relapse (1) |  |  |  |  |  |  |  |
| Methadone reduces crime and re-incarceration (2) |  |  |  |  |  |  |  |
| Methadone rewards criminals for being drug users (3) |  |  |  |  |  |  |  |
| Methadone prolongs addiction (4) |  |  |  |  |  |  |  |
| Methadone should be used to maintain clients who have opioid use disorder (5) |  |  |  |  |  |  |  |
| Methadone is more effective than non-pharmacological approaches (e.g. counseling) to retaining clients in treatment (6) |  |  |  |  |  |  |  |
| Methadone interferes with one’s ability to drive a car (7) |  |  |  |  |  |  |  |
| Methadone reduces or blocks the effect of heroin (8) |  |  |  |  |  |  |  |
| In Florida, it is difficult for a parent to regain custody of a child while the parent is treated with methadone (9) |  |  |  |  |  |  |  |
| People should be allowed to access methadone without counseling. (10) |  |  |  |  |  |  |  |
| Methadone prescribers should have a titration plan for each patient (15) |  |  |  |  |  |  |  |

Q5 To what extent do you agree with the statements below about**oral/sublingual buprenorphine (e.g. Suboxone)**provided by a health care practitioner for opioid use disorder treatment?

|  | Strongly disagree (1) | Somewhat disagree (2) | Neither Agree nor disagree (3) | Somewhat agree (4) | Strongly agree (5) | I don't know (6) | I choose not to answer (7) |
| --- | --- | --- | --- | --- | --- | --- | --- |
| Buprenorphine (e.g. Suboxone) reduces relapse (1) |  |  |  |  |  |  |  |
| Buprenorphine (e.g. Suboxone) reduces crime and re-incarceration (2) |  |  |  |  |  |  |  |
| Buprenorphine (e.g. Suboxone) rewards criminals for being drug users (3) |  |  |  |  |  |  |  |
| Buprenorphine (e.g. Suboxone) prolongs addiction (4) |  |  |  |  |  |  |  |
| Buprenorphine (e.g. Suboxone) should be used to maintain clients who have opioid use disorder (5) |  |  |  |  |  |  |  |
| Buprenorphine (e.g. Suboxone) is more effective than non-pharmacological (e.g. counseling) approaches to retaining clients in treatment (6) |  |  |  |  |  |  |  |
| Buprenorphine (e.g. Suboxone) interferes with one’s ability to drive a car (7) |  |  |  |  |  |  |  |
| Buprenorphine (e.g. Suboxone) reduces or blocks the effect of heroin (8) |  |  |  |  |  |  |  |
| In Florida, it is difficult for a parent to regain custody of a child while the parent is treated with buprenorphine (e.g. Suboxone). (9) |  |  |  |  |  |  |  |
| People should be allowed to access buprenorphine (e.g. Suboxone treatment) without counseling. (10) |  |  |  |  |  |  |  |
| Buprenorphine prescribers should have a titration plan for each patient. (13) |  |  |  |  |  |  |  |

Q6 To what extent do you agree with the statements below about **depot injection, extended-release naltrexone (Vivitrol)** provided by a health care practitioner for opioid use disorder treatment?

|  | Strongly disagree (1) | Somewhat disagree (2) | Neither Agree nor disagree (3) | Somewhat agree (4) | Strongly agree (5) | I don't know (6) | I choose not to answer (7) |
| --- | --- | --- | --- | --- | --- | --- | --- |
| Extended-release naltrexone (Vivitrol) reduces relapse (1) |  |  |  |  |  |  |  |
| Extended-release naltrexone (Vivitrol) reduces crime and re-incarceration (2) |  |  |  |  |  |  |  |
| Extended-release naltrexone (Vivitrol) rewards criminals for being drug users (3) |  |  |  |  |  |  |  |
| Extended-release naltrexone (Vivitrol) prolongs addiction (4) |  |  |  |  |  |  |  |
| Extended-release naltrexone (Vivitrol) should be used to maintain clients who have opioid use disorder (5) |  |  |  |  |  |  |  |
| Extended-release naltrexone (Vivitrol) is more effective than non-pharmacological approaches (e.g. counseling) to retaining clients in treatment (6) |  |  |  |  |  |  |  |
| Extended-release naltrexone (Vivitrol) interferes with one’s ability to drive a car (7) |  |  |  |  |  |  |  |
| Extended-release naltrexone (Vivitrol) reduces or blocks the effect of heroin (8) |  |  |  |  |  |  |  |
| In Florida, it is difficult for a parent to regain custody of a child while the parent is treated with is treated with extended-release naltrexone. (9) |  |  |  |  |  |  |  |
| People should be allowed to access extended-release naltrexone (Vivitrol) without counseling. (10) |  |  |  |  |  |  |  |
| Extended-release naltrexone (Vivitrol) providers should have a titration plan for each patient. (13) |  |  |  |  |  |  |  |

Q23 For the court in which you spend the MOST time, please mark the extent that each of the following is true about **methadone** **treatment** for opioid use disorder.

|  | Strongly disagree (1) | Somewhat disagree (2) | Neither Agree nor disagree (3) | Somewhat agree (4) | Strongly agree (5) | I don't know (6) | I choose not to answer (7) |
| --- | --- | --- | --- | --- | --- | --- | --- |
| No methadone providers are located near our court (1) |  |  |  |  |  |  |  |
| No trustworthy methadone providers are located near our court (2) |  |  |  |  |  |  |  |
| Clients lack financial resources to pay for methadone treatment (3) |  |  |  |  |  |  |  |

Q24 For the court in which you spend the MOST time, please mark the extent that each of the following is true about **oral/sublingual buprenorphine treatment** **(e.g. Suboxone)** for opioid use disorder.

|  | Strongly disagree (1) | Somewhat disagree (2) | Neither Agree nor disagree (3) | Somewhat agree (4) | Strongly agree (5) | I don't know (6) | I choose not to answer (7) |
| --- | --- | --- | --- | --- | --- | --- | --- |
| No oral buprenorphine (e.g. Suboxone) providers are located near our court (1) |  |  |  |  |  |  |  |
| No trustworthy buprenorphine (e.g. Suboxone) providers are located near our court (2) |  |  |  |  |  |  |  |
| Clients lack financial resources to pay for oral buprenorphine (e.g. Suboxone) treatment (3) |  |  |  |  |  |  |  |

Q25 For the court in which you spend the MOST time, please mark the extent that each of the following is true about **depot-injection, extended-release naltrexone treatment (Vivitrol)** for opioid use disorder.

|  | Strongly disagree (1) | Somewhat disagree (2) | Neither Agree nor disagree (3) | Somewhat agree (4) | Strongly agree (5) | I don't know (6) | I choose not to answer (7) |
| --- | --- | --- | --- | --- | --- | --- | --- |
| No extended-release naltrexone (Vivitrol) providers are located near our court (1) |  |  |  |  |  |  |  |
| No trustworthy extended-release naltrexone (Vivitrol) providers are located near our court (2) |  |  |  |  |  |  |  |
| Clients lack financial resources to pay for extended-release naltrexone (Vivitrol) (3) |  |  |  |  |  |  |  |

Q35 How would you best describe the county in which you spend the MOST time as a court staff member?

- Completely urban (1)
- Mostly urban (2)
- Mostly rural (3)
- Completely rural (4)
- I choose not to answer (5)

Q37 Is there anything else you would like the researchers to know about policies, attitudes, and barriers related to **medication-assisted treatment** (e.g. formulations of methadone, buprenorphine, naltrexone) for opioid use disorder? ______________________________
